# The impact of regional and neighbourhood deprivation on physical health in Germany: a multilevel study

**DOI:** 10.1186/1471-2458-10-403

**Published:** 2010-07-08

**Authors:** Sven Voigtländer, Ursula Berger, Oliver Razum

**Affiliations:** 1 Dept. of Epidemiology & International Public Health, School of Public Health, Bielefeld University, Universitätsstraße 25, 33615 Bielefeld, Germany

## Abstract

**Background:**

There is increasing evidence that individual health is at least partly determined by neighbourhood and regional factors. Mechanisms, however, remain poorly understood, and evidence from Germany is scant. This study explores whether regional as well as neighbourhood deprivation are associated with physical health and to what extent this association can be explained by specific neighbourhood exposures.

**Methods:**

Using 2004 data from the German Socio-Economic Panel Study (SOEP) merged with regional and neighbourhood characteristics, we fitted multilevel linear regression models with subjective physical health, as measured by the SF-12, as the dependent variable. The models include regional and neighbourhood proxies of deprivation (i.e. regional unemployment quota, average purchasing power of the street section) as well as specific neighbourhood exposures (i.e. perceived air pollution). Individual characteristics including socioeconomic status and health behaviour have been controlled for.

**Results:**

This study finds a significant association between area deprivation and physical health which is independent of compositional factors and consistent across different spatial scales. Furthermore the association between neighbourhood deprivation and physical health can be partly explained by specific features of the neighbourhood environment. Among these perceived air pollution shows the strongest association with physical health (-2.4 points for very strong and -1.5 points for strong disturbance by air pollution, standard error (SE) = 0.8 and 0.4, respectively). Beta coefficients for perceived air pollution, perceived noise and the perceived distance to recreational resources do not diminish when including individual health behaviour in the models.

**Conclusions:**

This study highlights the difference regional and in particular neighbourhood deprivation make to the physical health of individuals in Germany. The results support the argument that specific neighbourhood exposures serve as an intermediary step between deprivation and health. As people with a low socioeconomic status were more likely to be exposed to unfavourable neighbourhood characteristics these conditions plausibly contribute towards generating health inequalities.

## Background

Research concerned with contextual influences on health, that is the effect of regional and neighbourhood factors on individual health outcomes, and their interplay with compositional factors, comprising the individual characteristics of those living in a region or neighbourhood, has made considerable progress over the past two decades [1-3]. Studies increasingly measure specific characteristics of the social and built environment, use purposeful definitions of areas in contrast to relying on administrative boundaries and analyse specific outcomes rather than, for instance, all-cause mortality or self-rated health [4].

For Germany there have been only a few studies on contextual influences on health so far. However, trends of income inequality over the past 20 years provide evidence for an increase of social inequalities in Germany [5]. Furthermore, studies dealing with the spatial dimension of these inequalities demonstrate substantial and partly rising disparities in living conditions between neighbourhoods as well as between regions [6-11]. For instance, the interquartile range for unemployment quotas on regional level, defined by the German counties ("Kreise & kreisefreie Städte"), was 6.4% in 1995 (Min. = 4.1%, Max. = 22.8%) and increased to 8.1% in 2006 (Min. = 3.7%, Max. = 27.6%) [11].

The available studies in Germany, which link contextual factors with individual health outcomes, either focus on regional level or neighbourhood level factors. On regional level Queste, using an ecological study design, shows that all-cause mortality is strongly associated with the regional unemployment quota [12]. Kibele provides evidence that regional level factors, which she combines to a mortality context score for each region, are associated with mortality among German pensioners independently of compositional factors. For men an increase in the context score by one standard deviation is associated with a 1.09-fold increase in the mortality rate ratio; for women it is 1.05-fold [13]. The mortality context score applied by Kibele is a sex-specific additive score, weighted by the mortality effects of selected regional level factors which remain significant in a regression model. For men these are living space, unemployment quota and the share of employees with university degree; for women these are unemployment quota, the share of employees with university degree and the gross domestic product (GDP) per inhabitant. However, both studies do not account for neighbourhood conditions, and they also provide no insight into the association between regional characteristics and specific health outcomes. The multilevel study by Breckenkamp et al. analyses the association between the regional proportion of persons living in relative poverty and cardiovascular risk factors. The study includes only six selected study regions using baseline data of the German Cardiovascular Prevention Study (DHP), which was collected between 1984 and 1986 [14].

On neighbourhood level, several multilevel analyses have been conducted. Wolf shows that for the city of Cologne, physical health is worse in the lower-status urban districts [15]. Using 1999 national data from the German Socio-Economic Panel Study (SOEP), Pollack et al. find that, among others, perceived noise and perceived air pollution in the local environment mediate the relationship between housing tenure and self-rated health [16]. However, their focus is on tenure effects rather than neighbourhood effects. Apart from that they use a very general outcome measure, self-rated health. Three publications based on multilevel analyses stem from the Heinz Nixdorf Recall Study (HNR). This study covers the three industrial cities Bochum, Essen and Mülheim within the Ruhr area which is the largest conurbation in Germany. Two of the analyses indicate a small but significant association between neighbourhood deprivation, defined by the unemployment quota, and cardiovascular risk factors like smoking as well as coronary artery calcification (CAC) [17,18]. The third one provides evidence for an association between traffic exposure as measured by the distance to major roads and CAC [19]. Apart from distance to major roads, none of these three studies measures specific neighbourhood exposures. Kohlhuber et al., for instance, showed that perceived exposure to noise as well as air pollution are significantly related to income (odds ratio (OR) = 1.52 and OR = 1.67, respectively, for equal to or less than 50% of the median household income vs. more than 150%) [20].

Internationally, the discussion on the area deprivation-health relationship is more advanced than in Germany. For instance, besides defining neighbourhoods by taking different radii around a person's home [21,22], some studies propose and try to systematically observe and measure neighbourhood features ("Ecometrics") as well as measure selection and self-selection into areas [23-25]. Looking at the international body of research there are substantial differences regarding the outcomes, (specific) exposures, mediators and levels which are being studied as well as the methods applied why a comparison with the existing German studies is beyond the scope of this article.

In the study presented here we measure regional variation in physical health across the whole of Germany. We explore whether regional as well as neighbourhood deprivation are negatively associated with physical health, and whether this association can be explained by specific neighbourhood exposures related to physical health. Using 2004 data from the SOEP merged with regional and neighbourhood information, we fitted multilevel linear regression models with subjective physical health as dependent variable. The models include regional and neighbourhood proxies of deprivation (i.e. regional unemployment quota, purchasing power of the street section) as well as specific neighbourhood exposures, comprising stressors like perceived noise and perceived air pollution as well as perceived recreational resources. In addition, we take clustering of the outcome variable at the household level into account [26].

## Methods

### Data

Data for this study we obtained from the publicly available German Socio-Economic Panel Study (SOEP). The SOEP is a large longitudinal survey of private households that was started in 1984 and provides information on all household members [27].

In the analysis we included individuals aged 18 and above who took part in the survey in 2004. In 2004, a variety of perceived neighbourhood characteristics were collected in the household questionnaire. The SOEP investigators matched additional information based on the street section of the households (outlined below) that have been purchased from microm GmbH, a commercial data provider [28]. We also matched regional data for 2004 to the SOEP data set, which was provided by the Federal Office of Building and Regional Planning (BBR) at the level of the 439 German counties ("Kreise & kreisfreie Städte") [29].

### Variables

#### Outcome variable

We assessed physical health by the physical health component score (PCS) based on the SOEP version of the short form 12 questionnaire (SF-12). The SF-12 aims to measure health-related quality of life and consists of 12 items which are aggregated to eight subscales of which four consist of one item each and the other four of two items each. The PCS combines these eight subscales by principal component analysis. The four subscales with the highest factor loadings (FL) are: "general health" (FL: 0.789), "physical functioning" (FL: 0.857), "role physical" (FL: 0.779), and "bodily pain" (FL: 0.788). Thus the PCS summarizes different aspects of physical health into a single measure. The PCS is standardized to a mean of 50 and a standard deviation of 10, higher values indicating better physical health. Andersen et al. provide further information on the computation of the PCS [30].

#### Regional variables

The measures of regional deprivation, we used, comprise indicators of the job market and employment opportunities as well as prosperity, ethnical composition and health care provisioning. The following variables are included in our data set: the number of unemployed inhabitants as a proportion of the dependent labour force (unemployment quota); the number of jobs subject to compulsory social security contributions as a proportion of inhabitants aged 15 to 65 (employment quota); average monthly net income of private households per inhabitant in Euro; the GDP per inhabitant in Euro; tax revenue per inhabitant in Euro; the number of foreigners as a proportion of all inhabitants; the number of physicians per 100,000 inhabitants [29]. We used the variable employment quota in addition to the unemployment quota because its nominator does not include marginally employed persons ("geringfügig Beschäftigte"). Thus the employment quota can be seen as an indicator of better paid employment. Given the persisting socioeconomic disparities between former East Germany (German Democratic Republic, GDR) and West Germany (Federal Republic of Germany, FRG), which have been reunified in 1990, we also allowed for east-west differences by including an indicator variable "East Germany" as additional deprivation measure.

#### Neighbourhood variables

The neighbourhood characteristics include average purchasing power of the street section as a general measure of deprivation (or affluence). There are approximately 1.5 million street sections in Germany, as defined by the *microm GmbH*, containing on average 27 households. The variable "purchasing power" is based on official revenue statistics data ("Lohn- und Einkommensteuerstatistik"). However, *microm GmbH *does not publish further details on the construction of the variable [28,31]. As the SOEP investigators do not provide an identifier for the street section of the households and moreover only very few street sections are expected to have more than one household participating in the study this variable was regarded as a household level variable in the statistical analyses. The same applies for the following variables which were collected through the household questionnaire completed by the head of household. However, as these variables primarily characterise the neighbourhood environment in which the households are situated we still call them neighbourhood variables.

As the level of deprivation may correspond to the location of the neighbourhood we also included distance to the nearest city centre (graded on a six-point Likert scale from "residence in the city centre" to "60 km or more"), type of residential area ("mostly old houses", "mostly newer houses", "mixed area", "commercial area", "industrial area", "other") as well as housing type ("farm house", "detached one or two family house", "one or two family row house", "building with 3 to 4 flats", "building with 5 to 8 flats", "building with 9 or more flats", "high rise building", "other"). For type of residential area and housing type we retained the category "not specified" due to a large number of missing values.

In addition, we assessed specific neighbourhood characteristics which are a potential intermediary step between neighbourhood deprivation and physical health. These specific characteristics are perceived disturbance by noise and air pollution (both graded on a five-point Likert scale from "none" to "very strong") as well as the availability of recreational resources comprising perceived walking distance to public green space and public sports/leisure facilities (both graded on a four-point Likert scale from "less than 10 min." to "not existing/unavailable by foot"). For the latter two variables we again retained the category "not specified" due to a large number of missing values.

#### Sociodemographic variables

To control for differences in the composition of regions as well as households we also included sociodemographic characteristics such as age and gender. Persons with a migration background were defined on the basis of their or their parents' citizenship or country of birth. Socioeconomic status was assessed by the following variables: education according to the classification "Comparative Analyses of Social Mobility in Industrial Nations" (CASMIN) with the categories "still in school", "low" ("bis Hauptschule"), "intermediate" ("Abitur/Realschulabschluss"), "high" (Hochschulabschluss) and "not specified" [32]; unemployment based on the current labour force status; income as the net household income weighted by the modified OECD equivalence scale which we log-transformed to achieve a symmetric distribution [33,34]. Apart from that we created an indicator variable for single person households.

#### Health behaviour variables

Individual health behaviour was controlled for by including indicator variables for smoking ("currently yes" vs. "currently no") and for the frequency of sports, gymnastics or fitness training ("never" vs. "regularly/occasionally") as well as including the Body Mass Index (BMI) in three categories ranging from "less than 25 kg/m^2^" to "30 kg/m^2 ^or more".

### Statistical analyses

We fitted a series of multilevel linear regression models [35] including regional and neighbourhood characteristics to analyse the association between area deprivation and physical health. All models apply the following hierarchy: individuals - level 1, households - level 2, regions - level 3. All variables characterising the neighbourhood in which the households are situated were included at level 2 (households).

First, we estimated a baseline model (Model 1), containing no explanatory variables except age and gender, followed by a compositional effects model (Model 2), additionally including socioeconomic variables and household type. We then built a regional plus compositional effects model, which further added proxy measures of regional deprivation. Here we employed a backward selection on the regional variables (based on the Wald test) to exclude all variables with a p-value greater than 0.1 (Model 3) [36].

Second, we included proxy measures for neighbourhood deprivation at level 2 (Model 4). This model contains average purchasing power of the street section as well as factors associated with the location of the neighbourhood. We again used backward selection to exclude all neighbourhood variables with a p-value greater than 0.1. To test to what extent the association between neighbourhood deprivation and physical health can be attributed to specific neighbourhood exposures we further added the variables perceived noise, perceived air pollution, perceived distance to green space and sports/leisure facilities at level 2 (Model 5). In a final model we controlled for individual health behaviour by including smoking, sports participation and BMI (Model 6).

Modelling was carried out with MLwiN 2.10 [37]. All models were estimated using the iterative generalised least squares (IGLS) procedure.

## Results

A total of 21,521 SOEP members aged 18 or above living in 11,693 households within 436 regions participated in the survey in 2004. There were no respondents in 3 of the 439 regions of Germany. The average number of sample members per region was 49 (standard deviation (SD) = 55) while there were on average 187,929 inhabitants (SD = 218,813) living in a German region in 2004 [38]. Table 1 shows the characteristics of the study sample. Of all the respondents 96.5% completed the SF-12 and have valid information for their PCS. Table 1 also shows that sample members on average live in a street section with an average purchasing power of 36,160 €. Furthermore the proportion of persons exposed to presumably unfavourable neighbourhood conditions differs according to the type of neighbourhood characteristic. Around 20.0% of the respondents state that public green space and public sports/leisure facilities are more than 20 minutes of walking away or unavailable, whereas 6.6% and 3.8%, respectively, feel a strong or very strong disturbance by noise and air pollution. Table 2 presents the characteristics of the German regions. For instance, the mean regional unemployment quota is at 12.2% and it considerably varies between regions with a standard deviation of 5.8.

**Table 1:** Characteristics of the study sample (n = 21,521), wave 2004 of the German Socio-Economic Panel Study

Variable	Mean	SD	Proportion	Number
				

*Outcome variable*				

PCS (SD)	49.9	10.0		

No PCS information, % (number)			3.5	745

				

*Sociodemographic variables*				

Mean age in years (SD)	47.7	17.1		

Gender, % (number)				

Male			48.0	10,331

Female			52.0	11,190

Migration background, % (number)			15.2	3,261

Education, % (number)				

Still in School			1.6	355

Low			38.9	8,372

Intermediate			37.1	7,992

High			19.5	4,200

Not specified			2.8	602

Unemployed, % (number)			7.2	1,556

Single household, % (number)			13.4	2,890

Net equivalence income in € (SD)	22,469	22,637		

				

*Neighbourhood variables*				

Average purchasing power of street section in € (SD)	36,160	7,341		

Distance to nearest city centre, % (number)				

Residence in the city centre			10.0	2,147

Less than 10 km			24.7	5,317

10 to less than 25 km			27.7	5,972

25 to less than 40 km			15.2	3,261

40 to less than 60 km			10.6	2,285

60 km and more			11.1	2,383

Missing			0.7	156

Residential area, % (number)				

Mostly old houses			31.6	6,791

Mostly newer houses			43.2	9,305

Mixed area			22.0	4,736

Commercial area			0.5	110

Industrial area			0.4	94

Other			0.3	58

Not specified			2.0	427

Housing type, % (number)				

Farm house			3.2	691

Detached one or two family house			37.1	7,990

One or two family row house			17.5	3,775

Building with 3 to 4 flats			10.0	2,146

Building with 5 to 8 flats			17.1	3,678

Building with 9 or more flats			12.5	2,684

High rise building			1.2	252

Other			0.1	19

Not specified			1.3	286

Disturbance by noise, % (number)				

None			42.3	9,105

Little			37.9	8,155

Tolerable			12.9	2,770

Strong			5.0	1,072

Very strong			1.6	340

Missing			0.4	79

Disturbance by air pollution, % (number)				

None			47.0	10,112

Little			39.0	8,394

Tolerable			9.6	2,069

Strong			3.1	675

Very strong			0.7	156

Missing			0.5	115

Walking distance to public green space, % (number)				

Less than 10 min.			56.7	12,209

10 to 20 min.			22.5	4,846

More than 20 min.			7.1	1,529

Not existing/unavailable by foot			11.2	2,421

Not specified			2.4	516

Walking distance to public sports/leisure facilities, % (number)				

Less than 10 min.			39.2	8,434

10 to 20 min.			35.3	7,603

More than 20 min.			14.0	3,015

Not existing/unavailable by foot			8.7	1,862

Not specified			2.8	607

Notes: PCS, physical component score; SD, standard deviation. * All neighbourhood variables were gathered through the household questionnaire completed by the head of household or matched to the households based on their addresses.

**Table 2:** Characteristics of the regions* (n = 439) according to the Federal Office of Building and Regional Planning (BBR), 2004

Variable	Mean	SD
		

Unemployment quota in % (SD)	12.2	5.8

Employment quota in % (SD)	46.9	15.7

Monthly net income of private households per inhabitant in € (SD)	1,401	183.7

Gross domestic product per inhabitant in € (SD)	24,757	9,834

Tax revenue per inhabitant in € (SD)	446	169

Proportion of foreigners in % (SD)	6.9	4.7

Physicians per 100,000 inhabitants (SD)	153.3	52.9

Notes: SD, standard deviation. * All regional data refer to the level of the 439 German counties ("Kreise & kreisfreie Städte").

Table 3 shows the results of the regional three-level models nesting individuals in households in regions. In our baseline model for PCS (Model 1), 2.4% of the total variance is estimated to be at the regional level, i.e. the random PCS variation of 1.7 across regions divided by 71.9. The inclusion of compositional variables to the model reduces the unexplained variance at the regional level by 0.3 points (Model 2). Among the compositional variables age, less education as well as being unemployed are negatively associated with physical health while being male as well as a higher income show a positive association with physical health. After adding regional variables at level 3 and fitting the model by a backward selection of the regional variables, only the variable "East Germany" remains in the model, showing an overall reduction in PCS of 1.0 points (Model 3). The coefficients of the compositional variables remain rather stable. The unexplained variation at the regional level declines further by 0.2 points. It should be noted that adding the variable "unemployment quota" instead of the variable "East Germany" gives a similar model fit (-2*loglikelihood = 146,460). The reason is that regions with high unemployment quotas cluster in the East and those with low unemployment quotas cluster in the West (Table 4). Sensitivity analyses, excluding the 27 regions with 10 or less respondents, confirmed the robustness of the results of Model 3 (not shown).

**Table 3:** Regional models with fixed effects, random effects and standard errors for PCS in 2004

	Model 1 (baseline)		Model 2 (+ sociodemographic variables)		Model 3 (+ sociodemographic/ selected regional variables**)	
	**β (SE)**	**p-value**	**β (SE)**	**p-value**	**β (SE)**	**p-value**

						

**Fixed effects**						

Constant	49.37* (0.11)	< 0.001	51.62* (0.17)	< 0.001	51.94* (0.18)	< 0.001

						

*Level 1 (individuals)*						

Age	-0.31* (0.00)	< 0.001	-0.31* (0.00)	< 0.001	-0.31* (0.00)	< 0.001

Male	0.92* (0.11)	< 0.001	0.70* (0.11)	< 0.001	0.71* (0.11)	< 0.001

Migration background			-0.19 (0.18)	0.296	-0.31 (0.18)	0.084

Education						

High			Ref.		Ref.	

Intermediate			-1.85* (0.17)	< 0.001	-1.87* (0.17)	< 0.001

Low			-3.10* (0.18)	< 0.001	-3.21* (0.18)	< 0.001

Still in school			-3.92* (0.47)	< 0.001	-3.93* (0.47)	< 0.001

Not specified			-3.11* (0.38)	< 0.001	-3.18* (0.38)	< 0.001

Unemployed			-0.80* (0.23)	< 0.001	-0.73* (0.23)	0.001

						

*Level 2 (households)*						

Single household			0.25 (0.18)	0.150	0.23 (0.18)	0.185

Net equivalence income (log)			1.40* (0.12)	< 0.001	1.31* (0.13)	< 0.001

						

*Level 3 (regions)*						

East					-1.03* (0.21)	< 0.001

						

**Random variation**						

Level 1 (individuals)	57.97 (0.81)		57.63 (0.80)		57.69 (0.80)	

Level 2 (households)	12.20 (0.72)		9.87 (0.69)		9.84 (0.69)	

Level 3 (regions)	1.73 (0.25)		1.45 (0.23)		1.24 (0.21)	

						

-2*loglikelihood (number of cases)	147,201 (20,775)		146,480 (20,775)		146,456 (20,775)	

Notes: PCS, physical component score; β, beta coefficients; SE, standard errors. * significant at the 5% level using the Wald test. ** Regional variables were selected using backward selection based on Wald test, p-values > 0.1.

**Table 4:** Clustering of regional unemployment quotas in 2004 into East and West

	West	East*	Row total
Number of regions with an UQ ≥ 15%, (proportion)	11 (2.5%)	105 (23.9%)	116 (26.4%)

Number of regions with an UQ < 15%, (proportion)	315 (71.5%)	8 (1.8%)	323 (73.6%)

Column total	326 (74.3%)	113 (25.7%)	439 (100.0%)

Notes: UQ, unemployment quota. * including Berlin. Own table, according to the Federal Office of Building and Regional Planning (BBR).

Table 5 shows the results of the regional and neighbourhood characteristics models. To compare the likelihoods we excluded cases with missing values except for the variables for which we retained the category "not specified" due to a large number of missing values. Model 4 resulted from backward selection of the neighbourhood variables "purchasing power of the street section", "distance to the nearest city centre", "type of residential area" and "housing type". Purchasing power shows a highly significant association to PCS while the other neighbourhood variables did not remain in the model. An increase in purchasing power by 10,000€ is associated with a 0.5 point increase in PCS. Adding neighbourhood variables attenuates the association of the dummy variable "East Germany" with PCS from -1.0 to -0.5 points.

**Table 5:** Regional and neighbourhood characteristics models with fixed effects, random effects and standard errors for PCS in 2004

	Model 4^a^ (sociodemographic/selected regional and neighbourhood variables**)		Model 5^a^ (+ specific neighbourhood variables)		Model 6^a^ (+ specific neighbourhood/health behaviour variables)	
	**β (SE)**	**p-value**	**β (SE)**	**p-value**	**β (SE)**	**p-value**

						

**Fixed effects**						

*Level 1 (individuals)*						

Smoking					-0.24 (0.13)	0.065

No sports					-1.48* (0.13)	< 0.001

BMI in kg/m^2^						

< 25.0					Ref.	

25.0- 29.9					-0.93* (0.13)	< 0.001

≥ 30.0					-3.42* (0.18)	< 0.001

						

*Level 2 (households)*						


Purchasing power in 1,000 €	0.05* (0.01)	< 0.001	0.04* (0.01)	0.001	0.03* (0.01)	0.015

Disturbance by noise						

None			Ref.		Ref.	

Little			-0.37* (0.17)	0.029	-0.37* (0.17)	0.027

Tolerable			-0.66* (0.24)	0.007	-0.65* (0.24)	0.007

Strong			-0.71* (0.35)	0.042	-0.64 (0.34)	0.065

Very strong			-0.61 (0.61)	0.317	-0.64 (0.59)	0.283

Disturbance by air pollution						

None			Ref.		Ref.	

Little			-0.39* (0.17)	0.021	-0.42* (0.17)	0.013

Tolerable			-0.95* (0.27)	< 0.001	-0.98* (0.27)	< 0.001

Strong			-1.53* (0.43)	< 0.001	-1.59* (0.42)	< 0.001

Very strong			-2.36* (0.85)	0.005	-2.19* (0.83)	0.009

Walking distance to public green space						

Less than 10 min.			Ref.		Ref.	

10 to 20 min.			-0.21 (0.17)	0.203	-0.18 (0.16)	0.269

More than 20 min.			-0.94* (0.27)	< 0.001	-0.82* (0.27)	0.002

Not existing/unavailable by foot			-0.71* (0.23)	0.002	-0.63* (0.22)	0.004

Not specified			-0.55 (0.47)	0.251	-0.48 (0.47)	0.308

Walking distance to public sports/leisure facilities						

Less than 10 min.			Ref.		Ref.	

10 to 20 min.			-0.32* (0.16)	0.038	-0.30 (0.15)	0.051

More than 20 min.			-0.72* (0.21)	0.001	-0.70* (0.21)	0.001

Not existing/unavailable by foot			-0.74* (0.26)	0.004	-0.63* (0.25)	0.012

Not specified			-2.30* (0.44)	< 0.001	-2.34* (0.43)	< 0.001

						

*Level 3 (regions)*						

East	-0.51* (0.24)	0.035	-0.39 (0.24)	0.101	-0.37 (0.23)	0.119

						

**Random variation**						

Level 1 (individuals)	57.76 (0.81)		57.62 (0.81)		56.71 (0.79)	

Level 2 (households)	9.82 (0.69)		9.31 (0.68)		8.38 (0.66)	

Level 3 (regions)	1.16 (0.20)		1.09 (0.20)		1.01 (0.19)	

						

-2*loglikelihood (number of cases)	144,387 (20,481)		144,204 (20,481)		143,657 (20,481)	

Notes: PCS, physical component score; β, beta coefficients; SE, standard errors; BMI, Body Mass Index. ^a ^adjusted for age, gender, migration background, education and unemployment at level 1 as well as household type and net equivalence income (log) at level 2. * significant at the 5% level using Wald test. ** Neighbourhood variables were selected using backward selection based on the Wald test, p-values > 0.1.

In further sensitivity analyses (not shown) we additionally included municipality size (graded on a seven-point Likert scale) as well as community type after Boustedt [39] in Model 4 but none of these variables was significantly associated with PCS.

The inclusion of specific neighbourhood exposures further attenuates the relationship between the general measures of deprivation (purchasing power, "East Germany") and PCS (Model 5), the latter one becoming insignificant. Among the specific exposures, perceived air pollution shows the steepest gradient, and all categories have a significantly negative coefficient compared to the reference category "none" (i.e. beta coefficient (β) = -2.4 points for very strong and β = -1.5 points for strong disturbance by air pollution, standard error (SE) = 0.8 and 0.4, respectively). For the variable "perceived walking distance to public green space", for instance, there is no clear gradient with the coefficient for the category "not existing/unavailable by foot" being smaller than those for the category "more than 20 min." (β = -0.7 vs. β = -0.9 points). Including deprivation measures (Model 3 and 4) as well as specific neighbourhood exposures (Model 5) lowers the beta coefficients of the socioeconomic variables compared to Model 2, i.e. the coefficients for low education and the logarithm of the household income diminish from -3.1 to -2.5 and 1.4 to 0.9, respectively. Adjusting for measures of individual health behaviour considerably improves the model fit (likelihood ratio test: p < 0.001) but it does not substantially change the coefficients of the specific neighbourhood characteristics (Model 6). This is in particular true for the perceived distance to recreational resources after including individual sports participation (β = -1.5 points for no sport participation, SE = 0.1) in the regression models.

## Discussion

### The impact of regional and neighbourhood deprivation on physical health

This study finds a significant association between regional deprivation and physical health, as measured by the SF-12, for a national sample of German households in 2004. Including neighbourhood deprivation, measured by the average purchasing power of the street section, attenuates this association and shows that neighbourhood deprivation is more strongly related to physical health. This relation is independent of compositional factors like age, gender and income.

Specific features of the neighbourhood environment explain parts of the association between deprivation and physical health even after controlling for individual health behaviours like smoking. Of the specific neighbourhood exposures, perceived air pollution shows the strongest association with physical health, possibly affecting physical health through increasing the risk of respiratory and cardiovascular diseases. There is also a distinct independent association between the perceived distance to recreational resources and physical health. This supports the argument that specific neighbourhood exposures act as an intermediary step between deprivation and health. As people with a lower socioeconomic status are more likely to be exposed to unfavourable neighbourhood conditions, it is plausible that these conditions further contribute towards generating health inequalities [19]. As expected, individual factors like age, education and income explain most of the inter-individual variability in physical health.

Individual sports participation does not seem to be the mechanism linking the availability of recreational resources to physical health. The reason is that including individual sports participation in our regression models does not change the association between the perceived distance to recreational resources and physical health.

The regional clustering of physical health is comparatively small with around 2.4% of the total variance in the baseline model, which adjusts for age and gender. The fact that the indicator variable "East Germany" is the regional factor having the strongest association with physical health suggests that in 2004, clustering of physical health has been predominantly in the East and the West. This is probably due to the socioeconomic disparities between regions in the former GDR and regions in the former FRG which are still apparent in 2004. For instance, regions with high unemployment quotas cluster in the East and those with low unemployment quotas cluster in the West. Our alternative model adding the variable "unemployment quota" instead of the indicator variable "East Germany" shows a similar model fit.

In line with previous studies we find a significant association between health and both regional as well as neighbourhood deprivation on top of individual factors like age, education, income and individual health behaviour that explain most of the inter-individual variability in health [13-18]. People with a low socioeconomic status were also more likely to be exposed to unfavourable neighbourhood conditions like air pollution [16,19]. However, this study goes beyond previous studies in that it presents national results across different spatial scales for the specific outcome physical health. Our results also suggest that only taking the regional level into account, as done by some studies [12,13], misses substantial disparities regarding neighbourhood deprivation. It would certainly be an advantage to use more objective measures of specific neighbourhood exposures like Dragano et al. did regarding traffic exposure [19]. However, their measure "distance to major roads" is a very crude indicator for the actual exposure to air pollution. In line with Maas et al. we do not find evidence that sports participation is the mechanism linking the availability of recreational resources to physical health [22].

### Strengths and limitations

Major strengths of this study are that it focuses on physical health and relates it to deprivation across different spatial scales using a large, national sample of German households. In addition it also investigates the impact of specific features of the neighbourhood environment like perceived noise, perceived air pollution and perceived recreational resources which are presumably linked to physical health. Thus it is possible to estimate the extent of regional clustering of physical health as well as to explore possible pathways linking deprivation and health. By controlling for compositional factors including age, education, income and individual health behaviours, it was possible to measure the net association between contextual characteristics and physical health.

The data we used for this study also has some limitations. Due to the cross-sectional design it is not possible to clarify the direction of causality. It may, for instance, well be that less healthy people are disproportionally selected into deprived neighbourhoods with unfavourable exposures or that healthy people are disproportionally selected into wealthy regions [24]. Such a selection bias would result in overestimating the association between neighbourhood exposures and physical health. We are partly controlling for this kind of bias by including numerous compositional factors.

Concepts such as regional income inequality were not measured in our analyses while we did include other regional factors like employment opportunities as well as health care provisioning. However, income inequality is markedly lower in the East and thus cannot explain the negative coefficient of the dummy-variable "East Germany"[40].

The measurement of specific neighbourhood exposures is based on the household questionnaire which is completed by the head of household. Thus, the exposure level is self-rated which makes it liable to individual knowledge and socially patterned expectations [41]. Apart from that the head of household may also not consider neighbourhood exposures in his/her assessment which, for instance, might be important recreational features to other household members. Also, to quantify the exposure duration it would be necessary to have information on the time individuals spent in the neighbourhood. However, these shortcomings are likely to result in underestimating the association between neighbourhood exposures and physical health.

The association between purchasing power and physical health may be partly due to unobserved heterogeneity of households as well as street sections regarding monetary assets and lifestyle patterns which tend to cluster within households or neighbourhoods. However, including specific neighbourhood exposures in the model reduces the coefficient of purchasing power suggesting that a substantial part of the association between neighbourhood deprivation and physical health may be due to such factors as noise, air pollution as well as the absence of recreational features.

## Conclusions

This study highlights the difference regional and in particular neighbourhood deprivation make to the physical health of individuals based on a large dataset for the whole of Germany. Our results support the argument that specific neighbourhood exposures serve as an intermediary step between deprivation and health. Of these exposures perceived air pollution shows the strongest association with physical health while there is also a distinct independent association between perceived noise as well as the perceived distance to recreational resources and physical health. It is people with a lower socioeconomic status who are more frequently exposed to unfavourable neighbourhood conditions. This means that a number of people who wish to use such resources in order to improve or restore their physical health could not find them within their neighbourhood. From a political perspective further segregation should be avoided as it very likely increases socio-economic differences in neighbourhood exposures and thereby contributes towards health inequalities.

Future research should employ a longitudinal study design so that alternative explanations such as reverse causality as well unobserved heterogeneity can be excluded. Furthermore it is advisable to test for interactions in analyses of a specific exposure and to make more use of objective measures of neighbourhood exposures or at least to assess the validity of perceived exposures.

## Competing interests

The authors declare that they have no competing interests.

## Authors' contributions

SV had the initial idea for this paper which was subsequently modified in discussions with UB and OR. SV and UB conducted the data analysis. Results were interpreted by all three authors. SV wrote the initial draft of the manuscript which was revised by all authors. All authors read and approved the final manuscript.

## Pre-publication history

The pre-publication history for this paper can be accessed here:

http://www.biomedcentral.com/1471-2458/10/403/prepub
